# Waterborne Toxoplasmosis, Northeastern Brazil

**DOI:** 10.3201/eid1302.060686

**Published:** 2007-02

**Authors:** Jorg Heukelbach, Vanessa Meyer-Cirkel, Rômulo César Sabóia Moura, Márcia Gomide, José Ajax Nogueira Queiroz, Peter Saweljew, Oliver Liesenfeld

**Affiliations:** * Federal University of Ceará School of Medicine, Fortaleza, Ceará, Brazil; † James Cook University School of Public Health, Tropical Medicine, and Rehabilitation Sciences, Townsville, Queensland, Australia; ‡ Charité Medical School, Berlin, Germany; § Mandacaru Foundation, Fortaleza, Ceará, Brazil; ¶ Biomérieux, Nürtingen, Germany

**Keywords:** Toxoplasmosis, Toxoplasma gondii, epidemiology, prevalence, pregnancy, Brazil, dispatch

## Abstract

Two waterborne outbreaks of toxoplasmosis have been described recently in southern Brazil. We present data from a community-based study of pregnant women in northeastern Brazil. Consumption of homemade ice was the only variable associated with seropositivity (adjusted odds ratio, 3.1, 95% confidence interval, 1.53–6.24). Our results suggest water as a source of infection with *Toxoplasma gondii*.

*Toxoplasma gondii* is usually transmitted by consumption of food or water contaminated with oocysts from cat feces or soil or by eating undercooked meat that contains oocysts (*1*,*2*). Data from Canada and southern Brazil indicate that infection also occurs by drinking unfiltered water contaminated with oocysts (*3*–*6*). In Latin America, seroprevalence of immunoglobulin G (IgG) to *T*. *gondii* is generally high and ranges from 51% to 72% (*2*). In Brazil, factors predisposing for infection with *T*. *gondii* are not completely understood, and relatively little is known about the epidemiology of toxoplasmosis.

## The Study

This community-based cross-sectional study was undertaken in Cascavel Municipality, a typical semirural municipality ≈70 km south of Fortaleza, the capital of Ceará State in northeastern Brazil. In 2003, Cascavel had a population of 57,000. The main sources of income are fishing, agriculture, tourism, and the cashew nut industry.

Most (95%) of the pregnant women in Cascavel are registered in the Family Health Program and receive prenatal care from the public health system. All pregnant women at <26 weeks of gestation registered in this program from May to August 2003 were visited at home and asked to participate in the study. One female investigator interviewed the women with respect to demographic, socioeconomic status, and behavioral characteristics by using pretested structured questionnaires. Emphasis was given to the presence or ownership of animals, eating habits, soil contact and drinking water sources. The questionnaire was adapted from a study conducted in southern Brazil (*3*).

Serum samples were tested for IgM and IgG antibodies to toxoplasma by ELISA (Vidas, bioMérieux, Nürtingen, Germany). Women with positive IgG titers but negative IgM titers were considered latently infected. Women with positive titers both for IgG and for IgM were considered to possibly have recent infections and were further tested for avidity of IgG antibodies (Vidas, bioMérieux). Sensitivity and specificity of the Vidas test are 97.3% and 99.8, respectively (*7*). We were unable to rule out recent infections in women with low or intermediate IgG avidity.

Ethical approval for the study was obtained from the ethical review board of Cascavel Municipality. Before the study, community meetings were held in which the objectives of the study were explained. Informed written consent was obtained from all study participants. Women with possible recent infections and their newborns received free therapy and medical assistance.

Data were entered twice into a database by using EpiInfo version 6.04d software (Centers for Disease Control and Prevention, Atlanta, GA, USA) and checked for errors. Multivariate logistic regression with backward elimination was used to calculate adjusted odds ratios for the independent association between toxoplasma infection (defined as the presence of specific IgG antibodies) and possible risk factors. For logistic regression analysis, STATA version 7 software (Stata Corporation, College Station, TX, USA) was used.

A total of 231 pregnant women were identified during the study period, and all agreed to participate (median age 23 years, range 14–43 years). Of these women, 161 (69.7%, 95% confidence interval [CI] 63.3–75.6) had IgG antibodies against *T*. *gondi*. A total of 68% of women <25 years of age were seropositive. Prevalence was not significantly higher in older women than in women <25 years of age (p = 0.5). Five women (2.2%, 95% CI 0.7–5.0) had IgM antibodies; of these women, 3 (60%) had low-avidity IgG antibodies.

Bivariate analysis for factors associated with *T*. *gondii*–specific IgG showed that none of the demographic or socioeconomic variables were associated with infection. Other risk factors previously described, such as contact with cats or consumption of raw meat, were not associated with IgG seropositivity. In the logistic regression model, the only variable associated with IgG antibodies to toxoplasma was regular consumption of homemade ice (Table). Four (80%) of the 5 IgM-positive women regularly consumed these ices. This ice is made by people at home, is sold locally, and consists of tap water, artificial flavor, and sugar, frozen in small plastic bags.

**Table:** Multivariate logistic regression analysis of factors associated with infection with *Toxoplasma gondii* in pregnant women, northeastern Brazil

Independent variable	Adjusted odds ratio	95% Confidence interval	p value
Regular consumption of homemade ice	3.10	1.53–6.24	0.002
Having feral cats in yard	1.72	0.85–3.47	0.13
Being of low socioeconomic status*	0.94	0.77–1.14	0.5
Living on an unpaved street	0.50	0.23–1.07	0.07
Free-ranging chickens in yard	0.40	0.19–0.81	0.01
Consumption of cow milk	0.42	0.16–1.10	0.08
Consumption of cheese	0.47	0.25–0.90	0.02
Consumption of ice cream	0.59	0.31–1.11	0.10
Consumption of chicken	0.22	0.057–1.30	0.10

*According to an ordinal socioeconomic score from 0 to 10.

Using a commercial extraction kit (QIAGEN, Valencia, CA, USA), we extracted DNA from randomly chosen aliquots (1.5 mL) of >50 homemade ice samples obtained from local vendors in Cascavel and performed a standardized nested PCR assay (*T*. *gondii* B1 gene, sensitivity 1 parasite). Toxoplasma-specific DNA was not detected in any of these samples.

## Conclusions

The IgG prevalence of 70% found in this study is consistent with results of a study in Fortaleza in which 72% of pregnant and postpartum women were seropositive for IgG to toxoplasmosis (*8*). In our study population, prevalence did not increase with age, which indicated that in this setting most infections occur in childhood or adolescence. Only 2% of our study population had *T*. *gondii*–specific IgM antibodies.

Risk factors identified in other studies were not associated with toxoplasmosis in the typical semirural community in our study. A previous study from Brazil reported an outbreak of toxoplasmosis associated with the consumption of raw mutton (*9*). Other studies from south Brazil suggested that consumption of undercooked beef and working in a garden were risk factors (*3*,*10*). We did not find an association between consumption of raw meat and seropositivity, which may be because people in northeastern Brazil (unlike those in southern Brazil) do not eat undercooked or raw meat. None of our study participants reported eating undercooked meat.

Ownership of free-ranging chickens and consumption of cheese were negatively associated with toxoplasmosis. These 2 variables are associated with higher socioeconomic status in rural communities in northeastern Brazil. We cannot rule out that our results were confounded, even when we used a score to quantify socioeconomic status in multivariate regression analysis.

We found that homemade ice, which is stored in small plastic bags, was a possible risk for infection. However, toxoplasma DNA was not detectable in any of the ice samples. This finding does not rule out that the water was contaminated because identification of parasites in water requires large volumes. Drinking water (which is used for the preparation of this type of ice) was not a risk factor for infection. We cannot rule the possibility that the outer surface of the plastic bags in which the ice was packed was contaminated by oocysts from soil. These plastic bags are often opened by ripping them with the teeth, which may result in infection. Our results confirm the findings of Bahia-Oliveira et al. (*3*), who identified a marginal association between consumption of homemade ice stored in plastic bags and *T*. *gondii* infection.

Because our study was community based and included virtually all women who were pregnant during the study period, the results are highly representative for the pregnant population. However, our study has limitations. Because of the cross-sectional design, causal and temporal relationships are difficult to establish. Additionally, because few women were IgM positive, IgG positivity was used as a marker for toxoplasma infection. However, because IgG antibodies to toxoplama persist for years, many infections had probably been acquired some years ago, the environment and behavior patterns may have changed, and risks that are no longer present would not have been included.

In conclusion, toxoplasma infection in the study area was high in pregnant women. The study indicates that the pattern of risk factors for infection is different from that found in other studies. Future studies should show if these results are caused by chance or unknown confounders, or if the consumption of homemade ice has a direct association with infection with *T*. *gondii*.
